# The Distribution of Nanoclay Particles at the Interface and Their Influence on the Microstructure Development and Rheological Properties of Reactively Processed Biodegradable Polylactide/Poly(butylene succinate) Blend Nanocomposites

**DOI:** 10.3390/polym9080350

**Published:** 2017-08-09

**Authors:** Reza Salehiyan, Suprakas Sinha Ray, Jayita Bandyopadhyay, Vincent Ojijo

**Affiliations:** 1 DST-CSIR National Centre for Nanostructured Materials, Council for Scientific and Industrial Research, Pretoria 0001, South Africa; RSalehiyan@csir.co.za (R.S.); Jbandyopadhyay@csir.co.za (J.B.); VOjijo@csir.co.za (V.O.); 2 Department of Applied Chemistry, University of Johannesburg, Doornfontein 2028, South Africa

**Keywords:** reactively compatibilized clay-containing PLA/PBS blends, morphology development, non-linear rheological properties

## Abstract

The present work investigates the distribution of nanoclay particles at the interface and their influence on the microstructure development and non-linear rheological properties of reactively processed biodegradable polylactide/poly(butylene succinate) blend nanocomposites. Two types of organoclays, one is more hydrophilic (Cloisite^®^30B (C30B)) and another one is more hydrophobic (Betsopa^TM^ (BET)), were used at different concentrations. Surface and transmission electron microscopies were respectively used to study the blend morphology evolution and for probing the dispersion and distribution of nanoclay platelets within the blend matrix and at the interface. The results suggested that both organoclays tended to localize at the interface between the blend’s two phases and encapsulate the dispersed poly(butylene succinate) phase, thereby suppressing coalescence. Using small angle X-ray scattering the probability of finding neighboring nanoclay particles in the blend matrix was calculated using the Generalized Indirect Fourier Transformation technique. Fourier Transform-rheology was utilized for quantifying nonlinear rheological responses and for correlating the extent of dispersion as well as the blend morphological evolution, for different organoclay loadings. The rheological responses were in good agreement with the X-ray scattering and electron microscopic results. It was revealed that C30B nanoparticles were more efficient in stabilizing the morphologies by evenly distributing at the interface. Nonlinear coefficient from FT-rheology was found to be more pronounced in case of blends filled with C30B, indicating better dispersion of C30B compare with BET which was in agreement with the SAXS results.

## 1. Introduction

Bio-based polymers have attracted significant attention recently, owing to their biodegradability, environmental concerns, and capabilities as new alternatives to fossil fuel-based polymers [1,2]. Polylactide (PLA) is known to be one of the most available biodegradable polymers all, owing to its reasonable stiffness and thermal properties. However, it suffers from low toughness (~5 kJ/m^2^) characteristics, which restrict the range of its potential applicability, from food packaging to load-bearing [3,4]. Blending with other, higher impact strength, polymers has been previously suggested as a method for obtaining relatively tough nanocomposite materials [5]. To preserve the biodegradability of this nanocomposite material, other bio-based polymers, such as poly(ε-caprolactone) (PCL) [6], poly (butylene adipate-*co*-therephtalate) (PBAT) [7], poly [(butylene succinate)-*co*-adipate] (PBSA) [8] and poly(butylene succinate) (PBS) [9] have been blended with PLA to render their impact strength to the resulting nanocomposite material. Although polymer blending seems promising for broadening the range of material’s applicability, it should be noted that most polymers are thermodynamically immiscible, yielding phase-separated structures due to their low entropy of mixing ($\Delta_{mix}S$). Various phase-separated structures have been observed (e.g., sea-islands, lamellae, co-continuous structures, and salami) depending on different factors, such as processing conditions (e.g., mixing time, temperature, and intensity) and blend ratios. Alternatively, different morphologies can give rise to different characteristics. Therefore, morphology needs to be stabilized for obtaining desired properties. It has been shown that the addition of reactive processing agent can induce the compatibilization of the blends through branching and cross-linking interactions [10,11,12]. La Mantia et al. [10] revealed that styrene ethylene butylene styrene copolymer grafted with maleic anhydride (SEBS-*g*-MA) can compatibilize the blend of polypropylene/poly(ethylene terephthalate) PP/PET where finer morphologies obtained. Kumar et al. [11] studied the reactive compatibilization effects of glycidyl methacrylate (GMA) on the morphology of PLA/PBAT blends. They found that the formation of a random terpolymer at the interface could modify the interfacial properties and consequently compatibilize the blends due to the formation of chemical bonds between GMA and hydroxyl group of PLA and carboxyl group of PBAT. Al-Itry et al. [12] similarly found that Joncryl as a chain extender that can be used in PLA/PBAT blend to induce the branching/cross-linking reactions at the interface that led to the compatibilization of the corresponding blends.

Recently, inorganic solid nanoparticles as morphology stabilizers have received much attention, owing to their large specific area per unit volume and lower cost compared with available co-polymer compatbilizers [13,14,15,16,17,18]. Bhatia et al. [19] reported the compatibilization effects of Cloisite^®^30B (C30B) nanoclays on the PLA/PBS blends when the average droplet sizes reduced significantly upon C30B addition. Kumar et al. [11] found that Cloisite^®^20A (C20A) can intercalate and exfoliate into the PLA/PBAT matrices when GMA was used; which indicates that GMA can facilitate the exfoliation of clay particles into the blend. In another study, by Chen et al. [20] it was revealed that the compatibilization efficiency of the functionalized Cloisite^®^25A depended on the location of the nanoclay particles in a PLLA/PBS blend. When nanoclays were at PLLA phase, at low contents no size reduction was observed, while at high content some of the nanoclays were located at the interface and hindered the coalescence. This suggests that nanoclay localization can play an important role in controlling the morphology of the immiscible polymer blends. 

In addition, the structure of the polymers including polymer blends and nanocomposites has been reported to significantly affect their rheological and mechanical properties [21,22]. There have been many attempts to develop rheological methodologies and model viscoelastic behavior for obtaining the best structure-property correlation in polymer blends [23,24,25,26]. Over the last few years, rheological analysis based on large amplitude oscillatory shear (LAOS) flow has attracted significant attention owing to its high precision for predicting blends structure-property correlations [27,28,29]. A new technique, adapted to LAOS flows, is the Fourier transform (FT) rheology method, which allows to quantitatively probe non-linear rheological responses [30,31]. A correlation between the rates of droplet size reductions in polymer blends and the non-linear parameters from FT-rheology was found in previous studies [15,16,32,33]. For example, in the case of a (80/20) polypropylene/polystyrene (PP/PS) blend, it was found that hydrophobic nanoparticles (C20A; Cloisite^®^10A, C10A; fumed silica R202; SIPERNAT silica D17) trapped at the interface induce finer morphologies (stronger nonlinearity), while relatively hydrophilic nanoparticles (C30B; pristine Cloisite^®^Na^+^, CNa; fumed silica OX50) in the PS dispersed phase did not yield morphological improvements (weaker nonlinearity) [15,16]. In conclusion, while the effects of nanoparticles on the blend morphology have been studied, there has been no quantification of the distribution and dispersion of nanoclay particles at the interface and their influence on morphology development and rheological properties of immiscible polymer blend nanocomposites. 

The main objective of this work was to extensively investigate the extent of dispersion of two different organoclays and to quantify their effect on the microstructure development and rheology of reactively compatibilized PLA/PBS blends. Fourier Transform rheology was utilized for quantifying nonlinear rheological responses and for correlating the extent of dispersion as well as the blend morphological evolution, for different organoclay loadings. Small angle X-ray scattering (SAXS) and transmission electron microscopy (TEM) were used for probing the dispersion and distribution of nanoclay platelets within the blend matrix. The observations obtained using these methods supported the conclusions of rheological analysis.

## 2. Materials and Methods

### 2.1. Materials

**Figure 1:**
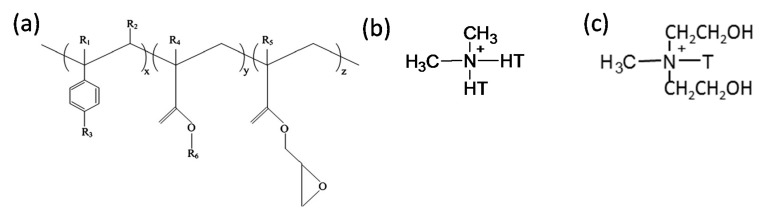
Chemical structure of (**a**) Joncryl^®^ ADR 4368, where x, y and z are between 1 and 20 R_1_, R_2_, R_3_, R_4_ and R_5_ are H, CH_3_, a higher alkyl group, or a combination of them; R_6_ is an alkyl group [5], (**b**) dimethyl dihydrogenated-tallow quaternary ammonium and (**c**) methyl tallow bis-2-hydroxyethyl quaternary ammonium, used in the modifications of South African bentonite and modified montmorillonite (MMT), respectively. T is tallow and it is a mixture of homologs C18, C16 and C14. HT is hydrogenated tallow.

The PLA used in the study was of an extrusion grade (commercially known as Terramac TE4000) and was obtained from Unitika Ltd., Kyoto, Japan. According to the supplier, PLA had a D-isomer content of approximately 1.2 to 2%, a density of 1.25 g/cm^3^, melting point of 170 °C, a glass transition temperature of 60 °C, and a melt flow index (MFI) in the 3–5 g/10 min range at 190 °C and 2.16 kg load. On the other hand, PBS (Bionolle 1001MD) was obtained from Showa Denko, Tokyo, Japan. It had a density of 1.26 g/cm^3^, glass transition temperature of −32 °C, MFI of approximately 3 g/10 min (at 190 °C and 2.16 kg load), and a melting temperature of 114 °C. The multi-functional oligomeric chain extender used in the study, Joncryl^®^ ADR 4368 CS, was kindly donated by BASF, Johannesburg, South Africa. The typical characteristics of this tailored styrene-acrylic oligomer with epoxy functions are listed in Table 1. Its chemical structure is shown in Figure 1a. It had a high number average functionality, *f*_n_ > 4.

The more hydrophobic organoclay used was Betsopa^TM^ (abbreviated as BET throughout manuscript), a commercially available organically modified montmorillonite (MMT) from our laboratory. BET is a South African calcium MMT modified with dimethyl dehydrogenated tallow quaternary ammonium surfactant (chemical structure, Figure 1b). Tallow is a mixture of homologs C18, C16 and C14. The amount of organic content in BET was measured using a thermogravimetric analyzer (TGA, Q500 TA Instrument, New Castle, DE, USA) and the surfactant content was found to be 28.6 wt % (data not presented here). On the other hand, C30B was used as a more hydrophilic organoclay, a commercial MMT (CNa) modified with methyl tallow bis-2-hydroxyethyl quaternary ammonium (chemical structure, Figure 1c). It was obtained from Southern Clay Products, Gonzales, TX, USA. According to the supplier (Southern Clay Products data sheet and our own TGA analysis, data not presented here), the C30B contained approximately 28 wt % surfactant. The quantity of surfactant in both organoclays was almost identical, and therefore, the surfactant content should not be considered as a significant independent variable in this work.

The interlayer spacing measured using X-ray scattering was 3.8 and 1.81 nm for BET and C30B, respectively. The solubility factors (δ) of surfactants used for the modification of MMTs, PLA, and PBS were roughly calculated on the basis of group contribution method of Fedors [34] and calculated values were 16.8, 21.5, 21.4, and 22.8 J^1/2^⋅cm^−3/2^ for surfactant used for BET, surfactant used for C30B, PLA, and PBS, respectively. The closure values of the polar solubility parameters for C30B, PLA, and PBS indicate that PLA and PBS matrices will have favorable enthalpic interaction with C30B than BET. In such scenario, we may expect that C30B will be localized mostly at the interface region, which lead to the finer morphology and improved properties of C30B-modified PLA/PBS blends. However, in the case of various organoclay modified immiscible blend nanocomposites, the results showed that a minimum interlayer spacing of organoclay was needed in order to have common intercalation of both polymer chains at the interface, and hence, improved thermal and mechanical properties of the blend nanocomposite [35].

### 2.2. Reactive Processing of Blends and Nanocomposites 

Before melt-extrusion, both PLA and PBS were dried overnight (15h) at 80 °C and 60 °C under vacuum, respectively. For this study, an optimized method of nanocomposite processing involved a two-step extrusion process. In the first step, PLA/PBS-Joncryl was processed in a co-rotating twin screw extruder (TE-30 Co-Rotating Twin Screw Extruder, Nanjing ONLY Extrusion Machinery Co. Ltd., Nanjing, China) at a screw speed of 120 rpm, and a feed rate of 4 kg/h. The temperature profile ranged from 160 °C to 190 °C along the 40 L/D screw profile (Diameter of the screw was 30 mm). The PLA/PBS ratio was maintained at 60:40, while 0.6% of the chain extender was used. This model composition was chosen based on our study on fractured-surface morphology and tensile property measurement. The results showed a substantial toughening of PLA with balance of modulus and strength when PLA reactively blended with 40 wt % PBS and 0.6 wt % Joncryl chain extender. The concentrations of Joncryl of <1 wt % were found to be below the threshold required for gelation in the PLA/PBS system, and therefore, it is expected that the 0.6 wt % Joncryl content used in the current study does not lead to network formation but rather just a long chain branched structures [5]. The second step involved incorporating the organoclays into the PLA/PBS/Joncryl blend, through the same twin screw extrusion. The screw speed was maintained at 150 rpm, while the feed rate was 4.4 kg/h. The temperature profile ranged from 120 °C to 185 °C along the screw profile. The organoclay concentration was such that the inorganic content was 1.5, 3, and 5 wt % in the final nanocomposites. The samples were coded: PLA/PBS/J/*x*%/BET or C30B, where *x* represented the percentage inorganic content in the sample, while J represented Joncryl (0.6 wt %). The processed samples were compression-molded, using a Carver laboratory press at 190 °C for 10 min, into test specimens, for further characterization.

### 2.3. Rheological Measurements 

Melt-rheological measurements were performed using a Physica MCR501 rheometer (Anton Paar, Austria) with 25-mm-diameter parallel plates under nitrogen environment. Small amplitude oscillatory shear (SAOS) tests were conducted, for frequencies ranging from low (0.1 rad/s) to high (100 rad/s), at a fixed strain of 0.5% (linear region) and temperature of 190 °C. Nonlinear rheological responses were acquired under LAOS flows combined with the Fourier Transform (FT)-rheology method, at a fixed frequency of 6.28 rad/s and strain amplitudes varying from 0.01 to 500% at 190 °C.

### 2.4. Morphological Analysis

The surface morphologies of the matrices of various samples were studied using a scanning electron microscope (SEM; JEOL JSM 7500F, Tokyo, Japan). Compression-molded samples were cryogenically fractured, and the resulting surfaces were coated with gold/palladium alloy and imaged at an accelerating voltage of 3 kV to minimize charging. The number average ($R_{n}$) and volume average ($R_{v}$) droplet sizes were calculated according to Equations (1) and (2), respectively. The radii of over 100 droplets were calculated for each sample from 3 different images, using image analysis software (ImageJ, National Institute of Health, Bethesda, MD, USA).

(1) $$R_{n}=\frac{\sum^{\text{​}}n_{i}R_{i}}{\sum^{\text{​}}n_{i}}\;$$

(2) $$R_{v}=\frac{\sum^{\text{​}}n_{i}R_{i}^{4}}{\sum^{\text{​}}n_{i}R_{i}^{3}}\;$$

where $n_{i}$ is the number of droplets with radius $R_{i}$.

Samples for TEM studies were prepared by removing a 1-mm^3^-volume sub-sample from the center of compression-molded samples from PLA/PBS/J with 1.5 and 5 wt % loading of C30B or BET, contrasted by immersion in 0.5% OsO_4_ overnight before trimming and sectioning using a Leica FC6 cryo-ultramicrotome (Leica, Wetzlar, Germany) at −80 °C. The collected sections were imaged at 200 kV using a JEOL JEM 2100 HRTEM (JEOL, Tokyo, Japan). Images were captured using a Gatan Ultrascan camera and Digital Micrograph software (Gatan, Pleasanton, CA, USA). 

### 2.5. SAXS Studies

SAXS experiments were performed using an Anton Paar SAXSess instrument, operated at 40 kV and 50 mA with point collimation geometry. The radiation used was a CuKα radiation with a wavelength of 0.1542 nm (PAN analytical X-ray source, Almelo, The Netherlands). Intensity profiles were obtained using a point-collimated SAXSess and recorded using a two-dimensional imaging plate. The samples were tilted by 90° with respect to the incident X-ray beam. A variostage sample holder with the tilt angle measurement set-up was used for this purpose. The sample-to-detector distance for the tilt angle was 260.24 mm and the radius of the detector curvature was 260 mm. The read-out angles were calculated from the pixel size, and the obtained *q* (scattering vector) scale was cross-checked by measuring silver behenate whose equidistant peak positions are known. All samples were exposed to X-rays for 30 min for determining the dispersion characteristics of silicate particles in the PLA/PBS/J blends. The compression-molded discs, approximately 1.7-mm-thick, were examined. To determine the dispersion/distribution characteristics of nanoclay platelets in the PLA/PBS blends, the corresponding 2D scattering patterns were analyzed using pi-profiles. 

## 3. Results and Discussion

### 3.1. Phase Morphology

**Figure 2:**
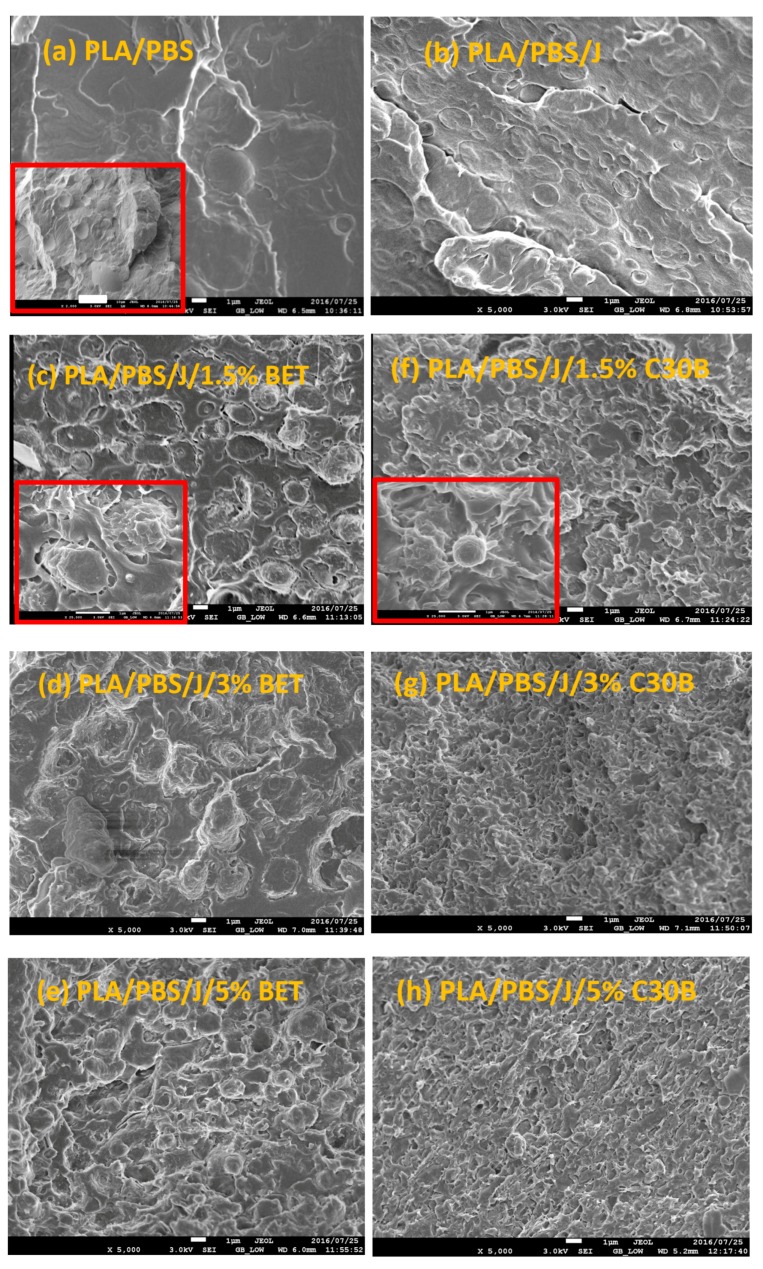
SEM images of (**a**) PLA/PBS, (**b**) PLA/PBS/J, (**c**–**e**) PLA/PBS/J/BET, and (**f**–**h**) PLA/PBS/J/C30B blends. The scale bars are 1 µm in all images. The scale bar in the inset image in (**a**) is 10 µm. The inset images in (**c**,**f**) are magnified showing two individual droplets in 1.5 wt % BET and C30B filled blends. BET is Betsopa™, C30B is Cloisite^®^30B, and J is Joncryl.

**Figure 3:**
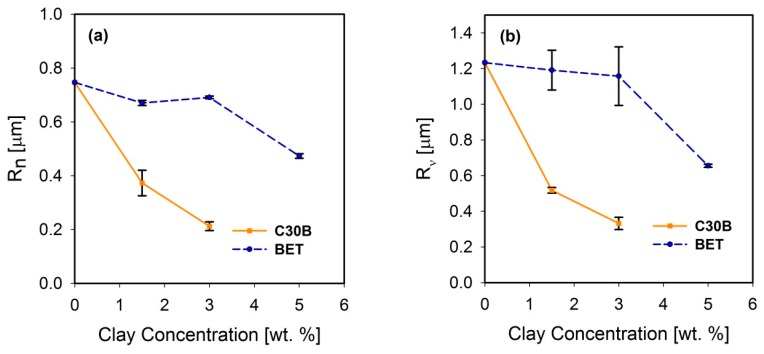
(**a**) Number average and (**b**) volume average droplet radii of the PLA/PBS/J blends filled with C30B and BET. The dashed and solid line plots represent the blends with BET and C30B, respectively. The morphology of the 5 wt % C30B-filled blend was not quite distinct for calculating droplet sizes. BET is Betsopa™, C30B is Cloisite^®^30B, and J is Joncryl.

Freeze-fractured SEM images of the neat blend and nanocomposite samples are shown in Figure 2. The number and volume average droplet radii are plotted in Figure 3a,b, respectively. Distinct droplet morphology can be observed for PLA/PBS (Figure 2a) and PLA/PBS/J (Figure 2b) blends. It can be seen that addition of Joncryl and the two types of organoclays (C30B and Betsopa™) has significantly reduced the dispersed PBS size.

**Figure 4:**
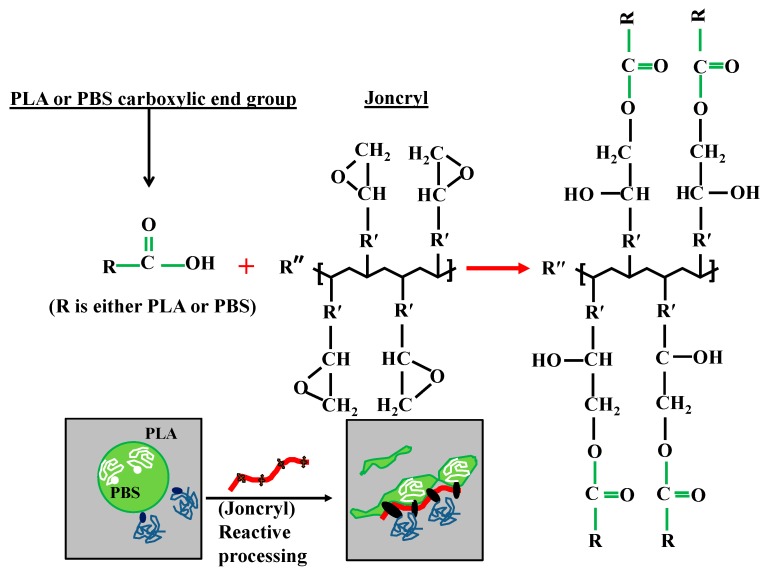
Schematic presentation of possible chemical reaction and formation of linear chain branched at the interface.

A significant size reduction can be seen in Figure 2b (and Figure 3) where 0.6 wt % Joncryl was added to the PLA/PBS blend. The efficiency of Joncryl in increasing the thermal stability and melt strength of PLA has also been discussed previously [5]. On the basis of published information, addition of Joncryl induces formation of long chain branched (LCB) (Figure 4) structures in PLA and PBS, which explains increasing viscosity of PLA/Joncryl and PBS/Joncryl. Moreover, the researchers [5] revealed that Joncryl could react with both PLA and PBS at the interphase and bring compatibility between the two phases. Meng et al. [36] revealed that epoxy groups in Joncryl could react with hydroxyl and carboxyl end groups of PLA, which led to a long chain branched structure. Chaiwutthinan et al. [37] also stated that epoxy group of the Joncryl could react with both hydroxyl and carboxyl group of the PLA and PBS in a PLA/PBS blend which resulted in a LCB structures. Similar phenomenon was observed in the works of Kumar et al. [11] and Al-Itry et al. [12] when they used Joncryl as an in situ reactive compatibilizer in PLA/PBAT blends. 

Therefore, on the basis of above discussion, it can be concluded that when Joncryl is added to PLA/PBS blends a copolymer is formed at the interface due to the chain linkages caused by the interaction of epoxy groups of Joncryl and the hydroxyl/carboxyl groups of the polyesters and the interface is immobilized and retards the film drainage between two approaching droplets, suppressing the coalescence of the droplets [38]. This leads to the improvement in the thermal and mechanical (particularly toughness) properties of compatibilized blends and these results are not reported here as the theme of this work is different. 

Later on, additional incorporation of organoclays reduces the droplet sizes; however, this size reduction effect is more significant in the case of C30B-filled blends in as much as the morphology for the 5 wt % C30B is no longer distinct sea-island morphology but behaves rather like a co-continuous morphology (Figure 2h). The inset images in Figure 2c,f show individual droplets in the blend with 1.5 wt % BET and C30B, respectively. It is clear that the dispersed phases in the 1.5 wt % BET filled blend of PLA/PBS/J/BET have rough surfaces, with polymer strands stretching at the interface. On the other hand, blend with 1.5 wt % C30B exhibits a smooth surface. This implies that there might be weaker adhesion between the PLA and PBS phases in the case of BET-filled blends, while C30B-filled blends yield lower interfacial tensions owing to the smooth interface, which is consistent with interfacial tension estimations discussed in Supplementary data. This is attributed to a better interaction of C30B with the PLA/PBS/J matrix, compared with that of BET. As shown in Figure 1, in BET, MMT is modified with a hydrophobic surfactant with no hydroxyl groups in its structure. On the other hand, the surfactant used in C30B contains hydroxyl groups in its structure, which enhances the enthalpic interaction with the PLA/PBS/J matrix. This conclusion also supported by the estimated δ values of various samples described in experimental section.

### 3.2. Dispersion and Localization of Nanoclay Platelets

**Figure 5:**
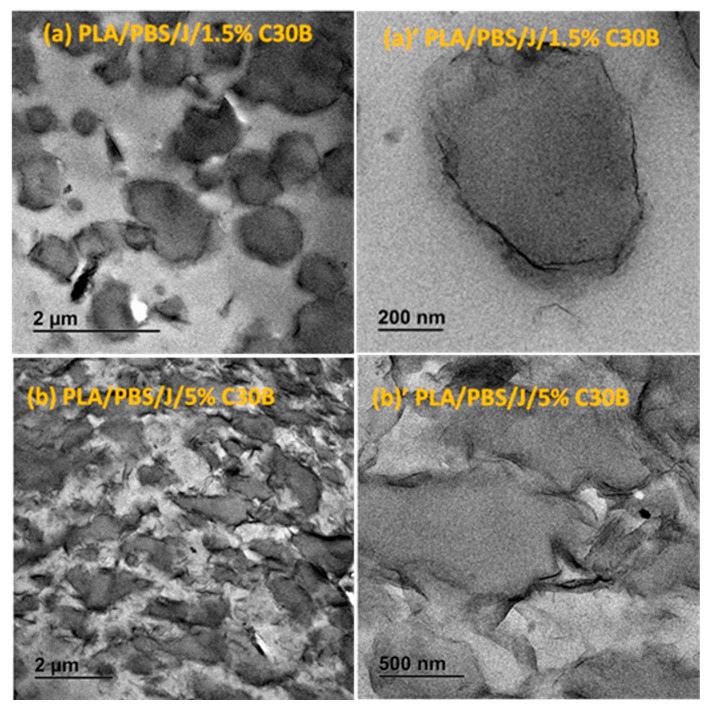
TEM images of the (**a**,**a’**) PLA/PBS/J/1.5%C30B and (**b**,**b’**) PLA/PBS/J/5%C30B blends. (**a’**) and (**b’**) are the high-magnification localized images of 1.5 wt % C30B (scale bar = 200 nm) and 5 wt % C30B (scale bar = 500 nm) blends. C30B is Cloisite^®^30B and J is Joncryl.

**Figure 6:**
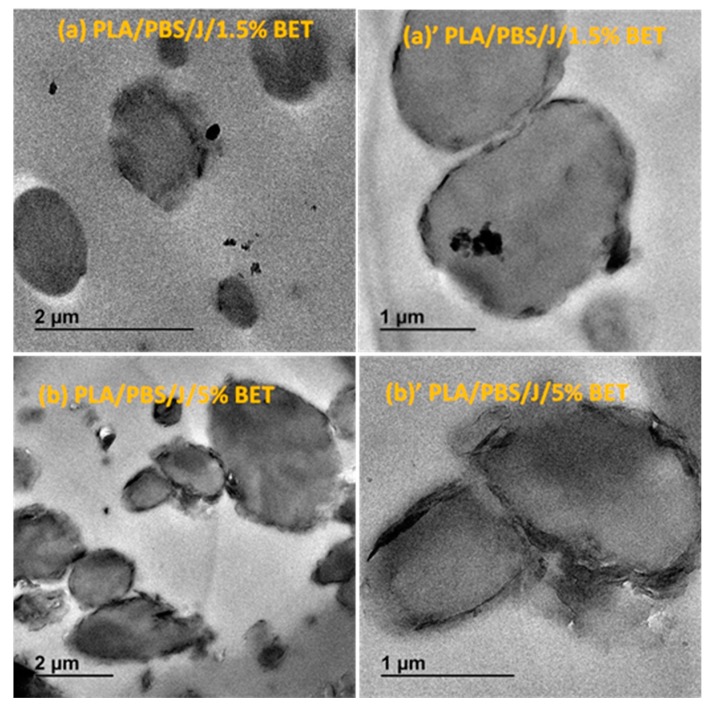
TEM images of the (**a**,**a’**) PLA/PBS/J/1.5% BET and (**b**,**b’**) PLA/PBS/J/5% BET blends. (**a’**) and (**b’**) are the high magnification localized images of 1.5 wt % BET (scale bar = 1 μm) and 5 wt % BET (scale bar = 1 μm) blends. BET is Betsopa™ and J is Joncryl.

The response of a blend to nanoclay incorporation relies on the nanoclay platelets localization and on the quality of dispersion within the blend matrix. TEM images in Figure 5 and Figure 6 elucidate the C30B and BET particles localizations, respectively, at low and high concentrations, revealing the stabilization mechanisms.

Overall from the TEM results, it can be seen that both types of organoclays mostly located at the PLA/PBS interface and encapsulated the PBS dispersed phases, accordingly suppressing the coalescence. For the C30B-filled system (Figure 5), the results show that C30B particles localized at the interface between PLA and PBS phases. On the other hand, some random agglomerations are clearly observed at PBS phase (see Figure 6) and at the interface in BET-filled blends, which is well supported by the SAXS analysis, as will be discussed in next section. This in turn yields droplets that are relatively larger than those in the C30B-filled blends. Random agglomerations in the dispersed phase and at the interface increase the viscosity of the PBS dispersed phase, making the breakup of the PBS droplets more difficult. Similar behavior has been reported for other systems [15,16,33,39,40]. Moreover, these findings are in accordance with the SEM results, which clearly indicate that 5 wt % C30B-filled blend has a complex co-continuous-like morphology while its corresponding BET-filled blend exhibits droplet morphology.

### 3.3. SAXS Analysis

**Figure 7:**
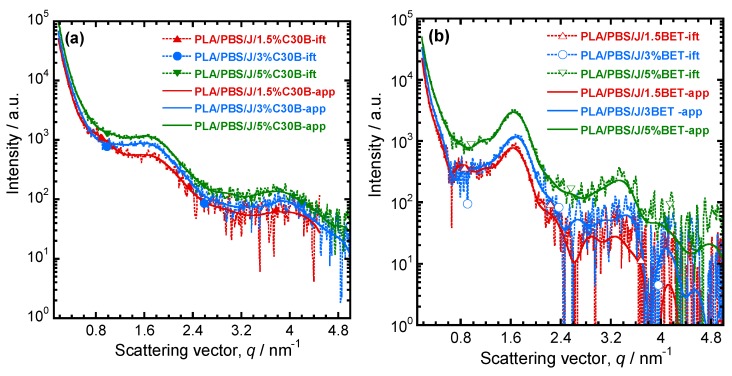
Background (scattering pattern of the PLA/PBS/J blend) subtracted scattering profiles of (**a**) PLA/PBS/J/C30B and (**b**) PLA/PBS/J/BET for different clay loadings. “ift” stands for the experimental scattering curve after background subtraction and “app” stands for the approximate scattering curves determined on the basis of GIFT analysis. BET is Betsopa™, C30B is Cloisite^®^30B, and J is Joncryl.

For determining the dispersion/distribution of nanoclay platelets in the PLA/PBS/J blend, the scattering pattern of PLA/PBS/J was considered as a background and subtracted from the nanoclay-filled blend nanocomposite patterns. After Porod extrapolation and subtraction of constant background, the scattering patterns were presented with “ift” extension. Figure 7a,b show the scattering curves for the blend nanocomposites containing C30B and BET, respectively. In Figure 7, sharp peaks appear in the BET-filled nanocomposites, compared with the C30B-filled nanocomposites. This indicates that parallel stacking of nanoclay platelets decreases significantly in the C30B-filled nanocomposites. However, in both systems, the parallel stacking increases with increasing nanoclay loading. 

The actual dispersion characteristics can be interpreted from the *d*-spacing, pair distance distribution function and the electron density profiles. The *d*-spacing increases when the polymer chains get intercalated in the clay galleries. To determine the *d*-spacing, the scattering angles were determined from the scattering vector according to Equation (3), where θ, *q*, and *λ* represent the scattering angle, the scattering vector and the wavelength of the incident X-ray, respectively.

(3) $$q=\frac{4\pi }{\lambda }\sin\theta$$

Now, according to Bragg’s law the *d*-spacing

(4) $$d=\frac{\lambda }{2\sin\theta }\;\left(\mathrm{for}\;n=1\right)$$

The scattering angles and the *d*-spacing values are listed in Table 2. It is noteworthy that the *d*-spacing values of pure C30B and BET are 1.8 and 3.8 nm respectively. In the case of the PLA/PBS/J/1.5%C30B, diffraction peaks appear at 1.2° and 2.4°, which correspond to *d*-spacing of 7.4 and 3.7 nm, respectively. The increase in the *d*-spacing indicates that polymer chains become intercalated in the C30B galleries. In PLA/PBS/J/3%C30B and PLA/PBS/J/5%C30B, diffraction peaks appear at 2.3° and 5.5°, which correspond to the *d*-spacing of 3.8 and 1.6 nm, respectively. This indicates that, although some polymer chains become intercalated in the nanoclay galleries, the *d*-spacing remains unaltered for certain stacks of C30B. This might be owing to the fact that certain C30B platelets disperse in PLA or PBS matrices and certain fraction is immobilized between the PLA and PBS interphases. On the other hand, in the case of BET-filled nanocomposites, a first order diffraction peak appears at 2.3°, which corresponds to the *d*-spacing of 3.8 nm. Since the *d*-spacing of BET remains unaltered in all of the nanocomposites, it is expected that BET platelets will remain stacked at the interfaces of the PLA/PBS blend. 

**Figure 8:**
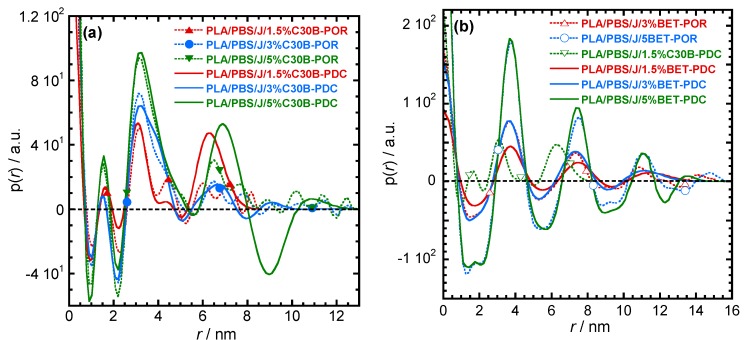
The pair-distance-distribution functions [p(r)] for (**a**) PLA/PBS/J/C30B and (**b**) PLA/PBS/J/BET for different clay loadings. “POR” denotes p(r) determined using the GIFT technique. The deconvolution of the approximate electron density distribution function provides a p(r) function, as denoted by “PDC”. BET is Betsopa™, C30B is Cloisite^®^30B, and J is Joncryl.

For more detailed understanding of the dispersion characteristics of the nanoclay platelets in the PLA/PBS/J matrix, the scattering patterns (presented in Figure 7) were analyzed using the Generalized Indirect Fourier Transformation (GIFT) technique (details can be found elsewhere [41]). According to this technique, the sum of the Fourier-transformed spline functions whose oscillations are restricted by Lagrange multipliers (λ_L_) yield an approximate scattering curve. This analysis yields the pair distance distribution function, p(r). It directly yields the probability of finding a pair of electron densities at a particular distance r. The GIFT technique requires to specify the number of spline functions and the upper limit of the largest particle dimension (*r*_max_). Initially, 40 spline functions were used, and then a particular λ_L_ was chosen for which the approximate scattering curve was similar to the experimental one. The values of *r*_max_ used for the GIFT analysis of different systems are listed in Table 2. The lowest *r*_max_ was obtained for PLA/PBS/J/1.5%C30B; *r*_max_ was higher for PLA/PBS/J/3%C30B and remained unaltered for PLA/PBS/J/5%C30B. The value of *r*_max_ remained almost the same for all BET-filled blend nanocomposites. In Figure 7, “app” denotes the approximate scattering curves. Figure 7 also shows that the approximate scattering curve matches well the experimental scattering curves (ift-patterns in Figure 7). Therefore, the p(r) function for electrons, from which the scattering curve was estimated, should be similar to the p(r) function that represents the experimental scattering patterns. The p(r) functions for different nanocomposites are presented as “POR” plots in Figure 8. The regions with opposite signs of different electron density yield negative contributions to p(r) and the correlation maxima (peak positions) represent the average radial distance to the next neighbour domains. Using a technique similar to the GIFT technique, it is possible to determine the electron density profiles for dispersed nanoclay platelets. The deconvolution of the approximate electron density distribution function yields the p(r) function, denoted by “PDC” in Figure 8. The similar trends of “POR” and “PDC” indicate that the approximate electron density profile captures the experimental scattering result. The locations of the correlation maxima for the nanocomposites are also listed in Table 2. It is evident from Figure 8 and Table 2, that in C30B-filled nanocomposites adjacent nanoclay platelets are at a shorter distance than those in BET-filled nanocomposites. It is noteworthy that the probability of finding a nanoclay platelet at a certain distance from another nanoclay platelet remains unaltered with increasing the organolay loading and is independent of the choice of organoclay. Therefore, the distribution of nanoclay platelet does not depend on the organoclay loading. However, the intensity of the scattering peak increases with increasing in organoclay loading. Therefore, it can be inferred that parallel stacking of nanoclay platelets increases with increasing organoclay loading. This effect is more prominent in BET-filled nanocomposites. The p(r) functions for the C30B-filled nanocomposites are quite different from those for the BET-filled nanocomposites. Four correlation maxima appear for PLA/PBS/J/1.5%C30B, while PLA/PBS/J/3%C30B and PLA/PBS/J/5%C30B exhibit three distinct correlation maxima. The trend of correlation maxima with increasing r_max_ indicates that the dispersion/distribution of C30B platelets changes as organoclay loading increases from 1.5 wt % to 3 wt %. Further increase in organoclay loading does not significantly affect the distribution of C30B platelets. This is in a good agreement with FT-rheology results, as will be discussed in the next section. The first correlation maximum presented in Figure 8a might be owing to the dispersed C30B platelets in the PLA matrix, while other correlation maxima likely represent the presence of overlapping nanoclay platelets at the PLA/PBS interface. On the contrary, stacked nanoclay platelets are uniformly distributed in BET-filled nanocomposites (Figure 8b).

**Figure 9:**
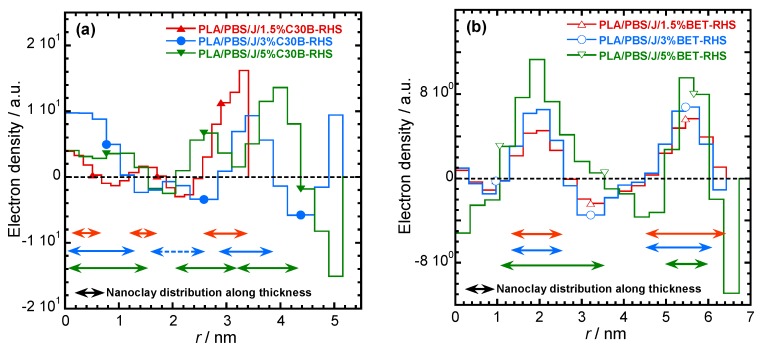
Electron density profiles for (**a**) PLA/PBS/J/C30B and (**b**) PLA/PBS/J/BET, for different clay loadings. BET is Betsopa™, C30B is Cloisite^®^30B, and J is Joncryl.

The electron density profiles for C30B- and BET-filled nanocomposites presented in Figure 9a,b, respectively. The figure shows the nanoclay platelets distribution along the thickness profile. According to Figure 9a smaller stacks of nanoclay platelets are present in PLA/PBS/J/1.5%C30B and co-continuity in dispersion and distribution of C30B platelets can be expected in PLA/PBS/J/3%C30B and PLA/PBS/J/5%C30B. This feature is absent in BET-filled nanocomposites (Figure 9b), where the stacked BET layers are slightly separated from each other, as observed in TEM images.

### 3.4. Linear Rheological Analysis

**Figure 10:**
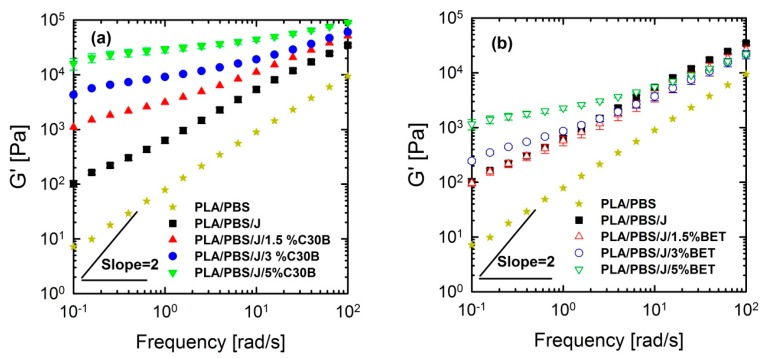
Storage (elastic) moduli of the PLA/PBS and PLA/PBS/J blends at various concentration of (**a**) C30B and (**b**) BET, at 190 °C. Strain amplitudes were small (0.5−1%) to ensure linear response. BET is Betsopa™, C30B is Cloisite^®^30B, and J is Joncryl.

Linear viscoelastic responses of the neat and nanoclay-filled blends were investigated by performing SAOS tests at 190 °C. Storage (elastic) moduli of the PLA/PBS and PLA/PBS/J blends and PLA/PBS/J blend filled with C30B and BET are shown in Figure 10.

**Figure 11:**
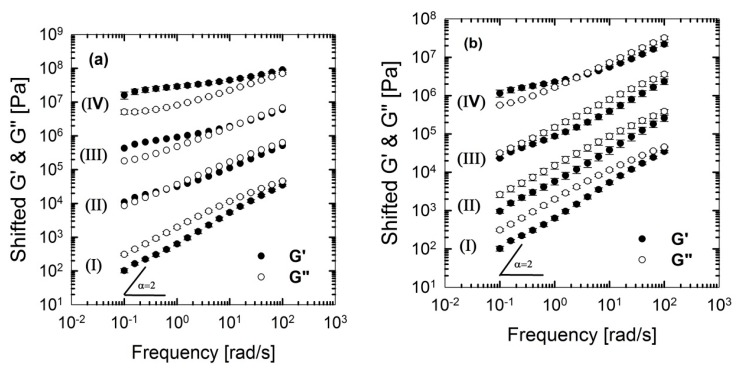
Storage (elastic), *G*’(ω) and loss (viscous), *G*’’(ω) moduli of the PLA/PBS/J blends, for various concentrations of (**a**) C30B and (**b**) BET, at 190 °C. Moduli are arbitrarily shifted by 10° (I = 0 wt % clay), 10^1^ (II = 1.5 wt % clay), 10^2^ (III = 3 wt % clay) and 10^3^ (IV = 5 wt % clay), respectively, for the sake of clarity. BET is Betsopa™, C30B is Cloisite^®^30B, and J is Joncryl.

From Figure 10 and Figure 11, it can be seen that PLA/PBS/J blend and blend nanocomposites exhibit non-terminal behavior at low frequencies. That is, all of blends disobey the classical behavior of homopolymers ($G\prime \propto \omega^{2}$) by exhibiting pseudo solid-like responses at low frequencies. This non-terminal behavior gradually becomes more prominent, whereas the slopes of the elastic moduli decrease with increasing organoclay loading. For high organoclay loadings, a plateau is reached, which is a strong indication of a highly elastic response (solid-like behavior). Enhancement of solid-like (non-terminal) behavior has been attributed to the emergence of filler networks [33] and compatibilization effects [42,43] in polymer nanocomposites and immiscible blends, respectively, where both are associated with the relaxation process of the polymer chains in nanocomposites and form (shape) relaxation process of the dispersed phase in immiscible blends. Compatibilization process in immiscible polymer blends promotes the terminal regions toward the plateau-like responses at low frequencies; this increases the longest relaxation time of the compatibilized blends and reduces dispersed phase size [15,44,45]. Furthermore, it demonstrates that Joncryl itself as a chain extender acts as a reactive compatibilizer for PLA/PBS blends when the blend’s elastic modulus *G*’(ω) increases following the introduction of Joncryl. As discussed earlier the addition of Joncryl could have introduced a LCB structure which alone can bring pseudo-solid-like behavior, and consequently, the non-terminal behavior. This is in a good agreement with morphological analysis observations (Figure 2 and Figure 3) of reduced droplet size following addition of Joncryl. These findings suggest that the blend morphology can be further stabilized by adding organoclays. However, this effect is more evident in the case of the C30B-filled blends, because as much as 1.5 wt % of C30B yields behavior similar to that obtained for 5 wt % BET-filled blend. Figure 11a,b clearly show that adding 1.5 wt % C30B to the PLA/PBS/J blend yields a crossover point, with both *G*’(ω) and *G*’’(ω) intersecting at low frequencies $\omega_{c}$ and this crossover point shifts to higher frequencies as the C30B loading increases, wherein *G*’(ω) dominated throughout the entire frequency range. On the other hand, incorporating 5 wt % BET into the blend introduces a crossover point at intermediate frequencies. Thus, C30B is expected to be more efficient in stabilizing the morphology of the blends, as was shown in previous sections for a more dramatic size reduction in case of the C30B-filled PLA/PBS/J blend. This is consistent with interfacial tension predictions from fitting results based on Palierne’s model (Figure S1a,b, Supporting data) where it was found that blends filled with C30B happened to have smaller interfacial tensions than those of BET-filled blends (See Table S1).

The above discussion suggests that different mechanisms might have been responsible for inducing solid-like behavior (increasing the blend elastic modulus) in the blends, in the following way: (i) initial reactive compatibilization of the blends with Joncryl; (ii) hydrodynamic effects of nanoclay particles; (iii) further morphological stabilization of the blends upon organoclay loadings.

### 3.5. Non-linear Rheological Analysis Based on LAOS Tests

**Figure 12:**
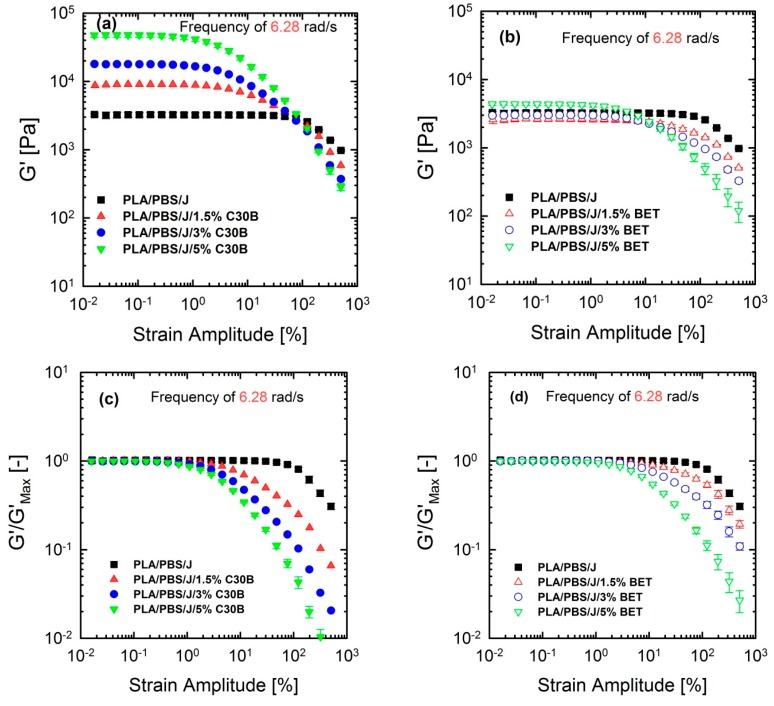
Storage (elastic) moduli *G*’($\gamma_{0}$) of the PLA/PBS/J blends, for various loadings of (**a**) C30B and (**b**) BET, at 190 °C, under the LAOS flow and a fixed frequency of 6.28 rad/s. (**c**,**d**) represent the normalized moduli of the their counterpart plots in (**a**,**b**). BET is Betsopa™, C30B is Cloisite^®^30B, and J is Joncryl.

Although linear rheological properties provide useful insights into the compatibilizer choice and the extent of dispersion, previous studies revealed that analysis based on non-linear rheological responses from LAOS tests could be more informative regarding the extent of stabilization and dispersion [15,46,47]. Previous studies revealed that 10 phr hydrophobic particles (R202 [16] and C20A [48]) exhibited higher *G*’(ω) at lower frequencies, compared with other hydrophobic particles (D17 [16] and C10A [48]) in a (80/20) PP/PS blend, although the blend morphology at this particular concentration was not consistent with linear rheological properties. This discrepancy was captured by non-linear rheological responses based on LAOS. Figure 12 shows the non-linear responses of the blends under a LAOS flow, where the strain amplitudes are swept from 0.01 to 500% at a fixed frequency of 6.28 rad/s.

Figure 12 shows that the dependence of dynamic storage moduli G’($\gamma_{0}$) on deformation, known as the Payne effect, increases with increasing organoclay loading. Loss moduli G”($\gamma_{0}$) demonstrated the similar trend (not shown here). The increase is much more prominent in the case of blends filled with C30B. Furthermore, Figure 12c,d reveal that adding organoclays enhances the strain-softening behavior. In other words, a critical strain amplitude, for which the modulus deviates from linearity, is shifted to lower amplitudes with increasing organoclay loading. Previous studies discussed the relationship between the strain-softening behavior and droplet deformability, and it was found that the higher the deformability of the droplets, the stronger the strain-softening and/or shear thinning behaviour [15,33,48,49]. On the other hand, this behavior in nanocomposites is owing to de-agglomeration and breakdown of networks of dispersed particles [33].

#### FT-Rheology

FT-rheology was coupled with LAOS experiments for quantifying non-linear responses and for acquiring more information to interpret the morphology of the nanoclay-filled blends. FT-rheology converts the stress signal into a series of odd-numbered higher harmonics of intensities, where the third relative harmonic ($I_{3/1}$) is usually the most informative [30,31]. The suitability of this approach to investigating and quantifying internal structure of materials has been frequently reported [47,50,51,52]. This method has been successfully utilized for detection of branching degrees of different materials [53,54]. Hyun and Wilhelm [53] established a new coefficient $Q\equiv I_{3/1}/\gamma_{0}^{2}$ based on FT-rheology and called the small-amplitude region of constant Q *zero-strain non-linear* coefficient $Q_{0}\left(\omega \right)\equiv \underset{\gamma_{0}\to 0}{\lim}Q\;\left(\omega ,\gamma_{0}\right)$.

**Figure 13:**
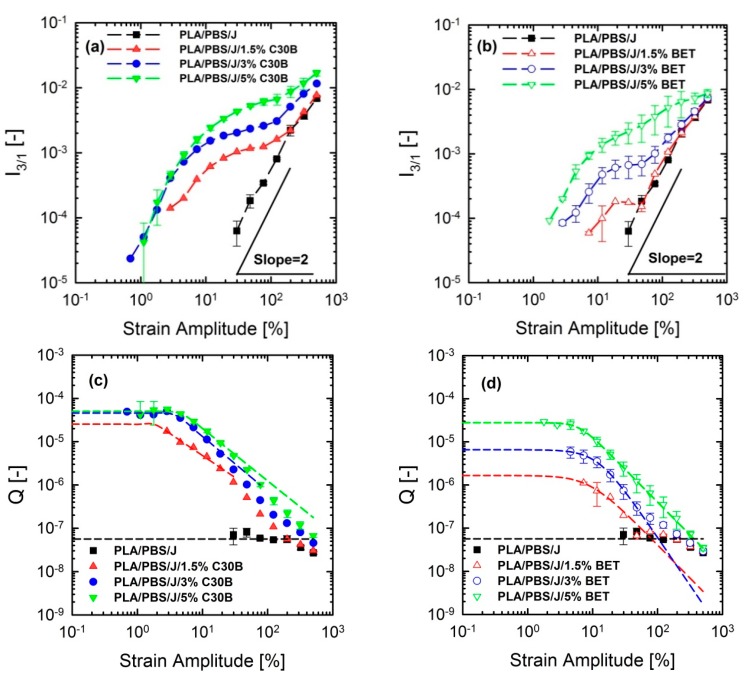
Normalized third relative ($I_{3/1}$) intensities of (**a**) PLA/PBS/J/C30B, (**b**) PLA/PBS/J/BET blends and $Q\equiv I_{3/1}/\gamma_{0}^{2}$ values of (**c**) PLA/PBS/J/C30B and (**d**) PLA/PBS/J/BET blends as a function of strain amplitude at 190 °C and fixed frequency of 6.28 rad/s. Q is fitted using a model in analogy with Carreau-Yasuda model $[Q=Q_{0}\left(1+{\left(C_{1}\gamma_{0}\right)}^{C_{2}}{)}^{\frac{C_{3}-1}{C_{2}}}\right]$ where $Q_{0}$ is the *zero-strain non-linear* coefficient and $C_{1},\;C_{2}\;$and $C_{3}$ are fitting parameters. BET is Betsopa™, C30B is Cloisite^®^30B, and J is Joncryl.

**Figure 14:**
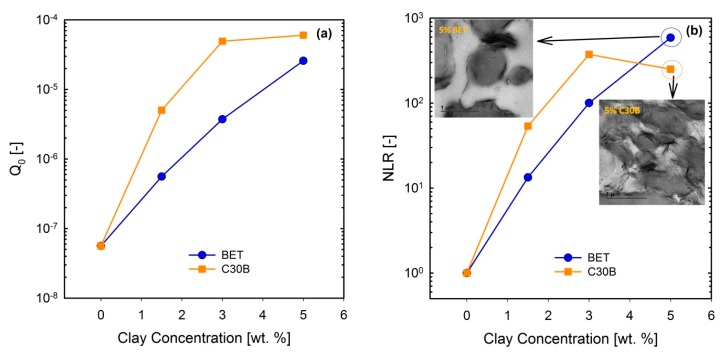
(**a**) $Q_{0}$ and (**b**) NLR values for the (60/40) PLA/PBS/0.6J blends filled with C30B and BET organoclays, for different loadings. Inset images in (**b**) are TEM images of 5 wt % blends of their corresponding blend. BET is Betsopa™, C30B is Cloisite^®^30B, and J is Joncryl.

Figure 13a–d show the normalized $I_{3/1}$ and their corresponding $Q\equiv I_{3/1}/\gamma_{0}^{2}$ parameters for the PLA/PBS/J/C30B and PLA/PBS/J/BET blend composites, for different organoclay loadings. The figure reveals interesting results, which are different from the linear regime results obtained from SAOS tests. Unlike the frequency sweep results in which C30B loading dramatically increased the moduli *G*’(ω) and *G*’’(ω) of the PLA/PBS/J blend, the results of FT-rheology analysis show that the addition of 5 wt % of C30B has no profound effect on $I_{3/1}$ especially in the MAOS regions corresponding to the $Q_{0\;}$ values, whereas both the 3 wt % and 5 wt % C30B blends exhibit similar $Q_{0}$ values. As discussed before $I_{3/1}$ is sensitive to microstructural changes in materials. Moreover, previous results revealed that FT-rheology could be used to correlate the extent of dispersion and compatibilization effects. Thus, it can be expected that 3 wt % is the optimized concentration for C30B-filled blends, and further addition of organoclays does not improve the blend morphology. This conclusion strongly agrees with the SAXS results, which indicate that dispersion for 3 wt % and 5 wt % is nearly the same. The SEM analysis results also yielded nearly the same morphologies at these two points. On the other hand, it can be seen that the intensities for the 1.5 wt % Betsopa™-filled blends are larger than that for the PLA/PBS/J blend in MAOS $Q_{0}$ regions, whereas SAOS results yield roughly similar responses. This is in a good agreement with morphological results indicating that droplets became smaller when 1.5 wt % BET is added to the system. Therefore, to put these observations in the same context, a normalized non-linear/normalized linear viscoelastic ratio (NLR) was used to correlate these internal structural changes to rheological properties of the structures. NLR is the normalized non-linear viscoelastic response obtained from LAOS experiments (FT-rheology), divided by the normalized linear viscoelastic response based on SAOS tests and is defined as follows [46].

(5) $$NLR=\frac{Q_{0}\left(\emptyset \right)/Q_{0}\left(0\right)}{\left|G^{*}\left(\emptyset \right)/G^{*}\left(0\right)\right|}\;$$

where ϕ is the filler concentration and $G^{*}$ is the complex modulus of the blend from SAOS experiments, acquired at a frequency of 6.28 rad/s. It has been reported that NLR is directly related to the extent of dispersion in nanocomposites [47,55,56,57]. Further, previous studies revealed that NLR is inversely proportional to the droplet size in immiscible polymer blends [15,16,32,48]. In a recent study on PP/PA nanocomposite blends, Sangroniz et al. [33] also found an inverse proportionality between $Q_{0}$ and droplet diameter. The values of $Q_{0}$ and their corresponding NLR values for the PLA/PBS/J blends filled with C30B and BET are plotted in Figure 14a,b, respectively.

Current results imply that $Q_{0}$ and NLR increase more rapidly in the C30B-filled blends up to 3 wt % than in the BET-filled blends ($NLR_{C30B-blends}>NLR_{BET-blends}$), while at 5 wt % C30B the increase is halted. Previous study revealed that NLR reflects phase morphology and extent of dispersion [16]. The present results can be attributed to both morphology and dispersion quality of the blends, at this particular concentration. This is supported by the SAXS results, which indicate that the extent of dispersion in the C30B-filled blends is rather higher than that in the BET-filled blends, manifested as a larger inter-particle distance in the C30B-filled blends. In addition, the levelling off phenomenon in $Q_{0}$ is nicely captured by SAXS, demonstrating similar dispersion qualities and no morphological improvements in TEM and SEM results. Thus, the results in Figure 14a ($Q_{0}\;\mathrm{vs}.\;clay\;concentration$) seem promising to discuss the differences in the extent of dispersion. Interestingly, Figure 14b (NLR vs. clay concentration) shows that NLR for the 5 wt % BET-filled blend is slightly higher than that for the 5 wt % C30B-filled blend, and this could be attributed to the different morphologies of the blends. Inset in Figure 14b shows that the morphology of the 5 wt % C30B-filled blend nanocomposite is no longer a sea-island morphology; rather, this blend exhibits a co-continuous structure. On the other hand, as shown in the top-left inset image in Figure 14b, the morphology of the 5 wt % BET-filled blend is still droplet morphology, although with large agglomerations. Thus, it appears that the results in Figure 14b better reflect the morphological changes, with the BET-filled blends exhibiting progressive improvement even at 5 wt %. However, overall the C30B-filled blends exhibit more stabilized morphologies. In general, the NLR values were inversely proportional to the morphology changes, especially in the case of C30B-filled blends.

## 4. Conclusions

Effects of two different types of organoclays on the structure and properties of PLA/PBS/Joncryl blends were studied for different nanoclay loadings. The obtained results indicated that Joncryl as a chain extender acts as reactive compatibilizer to trigger some initial morphological stabilizers by formation of a reactive copolymer at the interface, which hinders the film drainage at the interface. In addition, further organoclay loadings caused secondary size reduction by dispersed phase encapsulation at the interface, preventing excessive coalescence. Small amplitude oscillatory shear measurements revealed that organoclay loadings induced solid-like behavior when low frequency regions exhibited plateau moduli. C30B seemed to be more efficient by yielding remarkably larger elastic moduli associated with the relaxation process of droplets, compared with those of BET nanoclay platelets. It was found that both organoclays preferentially located mostly at the interface and PLA matrix. However, random agglomerations were found, within the blends when BET was used, which could explain smaller stabilization efficiency. This might be due to the less favorable enthalpic interaction between surfactant in BET and blend matrix, compared with that between the surfactant in C30B and blend matrix. SAXS results confirmed that C30B platelets dispersed better, compared with BET platelets. Finally, nonlinear rheological analysis based on large amplitude oscillatory shear measurements and FT-rheology results revealed that 3 wt % C30B is optimal for (60/40) PLA/PBS/0.6J blends, with non-linear viscoelastic ratio and $Q_{0}$ values levelling off above this loading. Interestingly, non-linear viscoelastic ratio values of the 5 wt % BET-filled blend were slightly larger than those of the 5 wt % C30B-filled blend, indicating that BET-filled blends still exhibit progress toward morphological improvement owing to droplet morphology at all concentrations. On the other hand, the 5 wt % C30B-filled blend exhibited morphological disruption. In summary, the results indicated that the main factor controlling the morphology of the blend is the favorable enthalpic interaction between polymer matrices and the surfactants used in the modification pristine MMTs. Moreover, rheological analysis based on large amplitude oscillatory shear and FT-rheology unveiled the role of organoclay as an interfacial modifier for immiscible polymer blend that could not be revealed from small amplitude oscillatory shear measurements.

## Figures and Tables

**Table 1:** Typical physical characteristics of Joncryl^®^ ADR 4368 CS.

Parameter	Value
Specific gravity, 25 °C	1.08
Molecular weight, *M*_w_	~6800 g/mol
Number average molecular weight, *M*_n_	3000
Glass transition temperature, °C	54
Epoxy equivalent weight	285 g/mol

**Table 2:** Results from SAXS analysis for various blend nanocomposite samples.

Sample	2θ/Degree			*d*-Spacing/nm			*r*_max_/nm	Distance (r/nm, Calculated from *.PDC Plots) to Find Neighbours			
	2θ_1_	2θ_2_	2θ_3_	*d*_1_	*d*_2_	*d*_3_					
PLA/PBS/J/1.5%C30B	1.2	2.4		7.36	3.68		8.6	1.5	3.2	4.6	6.3
PLA/PBS/J/3%C30B		2.3	5.5		3.84	1.61	13	1.5	3.2		6.7
PLA/PBS/J/5%C30B		2.4	5.5		3.68	1.61	13	1.5	3.2		6.9
PLA/PBS/J/1.5%BET		2.3	4.6		3.84		16.2		3.7	7.5	11.0
PLA/PBS/J/3%/BET		2.3	4.6		3.84		16		3.7	7.5	11.0
PLA/PBS/J/5%BET		2.3	4.6		3.84		16		3.7	7.5	11.0

The *d*-spacing values for C30B and BET are 1.84 nm and 3.84 nm, respectively, and the scattering patterns are not reported here. BET is Betsopa™, C30B is Cloisite^®^30B, and J is Joncryl.

## Supplementary Materials

The following are available online at www.mdpi.com/2073-4360/9/8/350/s1, Figure S1: Palierne model fits for (**a**) PLA/PBS and PLA/PBS/J blends and PLA/PBS/J/C30B and (**b**) PLA/PBS/J/BET blend nanocomposites. BET is Betsopa™, C30B is Cloisite^®^30B, and J is Joncryl, Table S1: Interfacial tension predicted by the Palierne model. BET is Betsopa™, C30B is Cloisite^®^30B, and J is Joncryl. The Palierne model might not be perfectly suitable for analysing these blends and blend nanocomposites; however, it could be used for the sake of estimation.
