# Supplementary material for: The Distribution of Nanoclay Particles at the Interface and Their Influence on the Microstructure Development and Rheological Properties of Reactively Processed Biodegradable Polylactide/Poly(butylene succinate) Blend Nanocomposites
Source: Polymers (Basel). 2017 Aug 9;9(8):350. doi: 10.3390/polym9080350 (PMC6418579; doi:10.3390/polym9080350)
Supplement: Supplementary file 1 [file polymers-09-00350-s001.docx]

Supplementary: The Distribution of Nanoclay Particles at the Interface and Their Influence on the Microstructure Development and Rheological Properties of Reactively Processed Biodegradable Polylactide/Poly(butylene succinate) Blend Nanocomposites

Reza Salehiyan^1^, Suprakas Sinha Ray^1,2^*, Jayita Bandyopadhyay^1^, Vincent Ojijo^1^

^1^ DST-CSIR National Centre for Nanostructured Materials, Council for Scientific and Industrial Research, Pretoria 0001, South Africa

^2^ Department of Applied Chemistry, University of Johannesburg, Doornfontein 2028, South Africa

* Correspondence: rsuprakas@csir.co.za; Tel.: +27-12-841-2388

Academic Editor: name

Received: date; Accepted: date; Published: date

Interfacial tension analysis

To examine the relative interfacial properties of the blends, the simplified emulsion model of Palierne [1] was used, as summarized in equations (S1) and (S2). Palierne model may be suitable for PLA/PBS blend but the situation is much complicated in the case of PLA/PBS/J system. Due to the reaction between PLA and PBS, both the dynamic moduli of PLA and PBS may change and the interfacial properties may be altered by the formation of co-polymer at the interface. The situation is more complicated in the presence of organoclay particles. TEM results already shown that most nanoclay platelets were located at the interface, however, few were dispersed in the matrices. Even if we assume that all nanoclay platelets are located at the interface, their influence on the flow field inside and outside the interface cannot resemble that of clean interface. Therefore, the Palierne model might not be perfectly suitable for analysing these blends and blend nanocomposites; however, it could be used for the sake of estimation.

| $G_{b}^{*}\left( \omega\right)=G_{m}^{*}\left( \omega\right)\frac{1+3\emptyset H^{*}(\omega)}{1-2\emptyset H^{*}(\omega)}$, | (S1) |
| --- | --- |
| $H^{*}\left( \omega\right)=\frac{4\left( \frac{\gamma}{R_{v}} \right)\left[ 2G_{m}^{*}\left( \omega\right)+5G_{d}^{*}\left( \omega\right) \right]+\left[ G_{d}^{*}\left( \omega\right)-G_{m}^{*}\left( \omega\right) \right][16G_{m}^{*}\left( \omega\right)+19G_{d}^{*}\left( \omega\right)]}{40\left( \frac{\gamma}{R_{v}} \right)\left[ G_{m}^{*}\left( \omega\right)+G_{d}^{*}\left( \omega\right) \right]+\left[ {2G}_{d}^{*}\left( \omega\right)+3G_{m}^{*}\left( \omega\right) \right][16G_{m}^{*}\left( \omega\right)+19G_{d}^{*}\left( \omega\right)]}$ | (S2) |

where $G_{b}^{*}\left( \omega\right)$, $G_{m}^{*}\left( \omega\right),$ and $G_{d}^{*}\left( \omega\right)$ are the complex moduli of the blend, the matrix and the dispersed phase at the angular frequency of $\omega,$ respectively. The quantities $\emptyset$, $R_{v},$ and $\gamma$ are the volume fraction of dispersed phase, the volume averaged droplet size of the dispersed phase and the interfacial tension, respectively. The Palierne model assumes a narrow dispersed phase distribution, with polydispersity below 2. Therefore, an attempt to estimate the interfacial tensions between the PLA and PBS phases by fitting the complex moduli of the blends using the Palierne model was made. The results of these fits are shown in Figures S1a,b. It was not possible to fit the Palierne model for blends with organoclays beyond 3 wt. % loading.

| 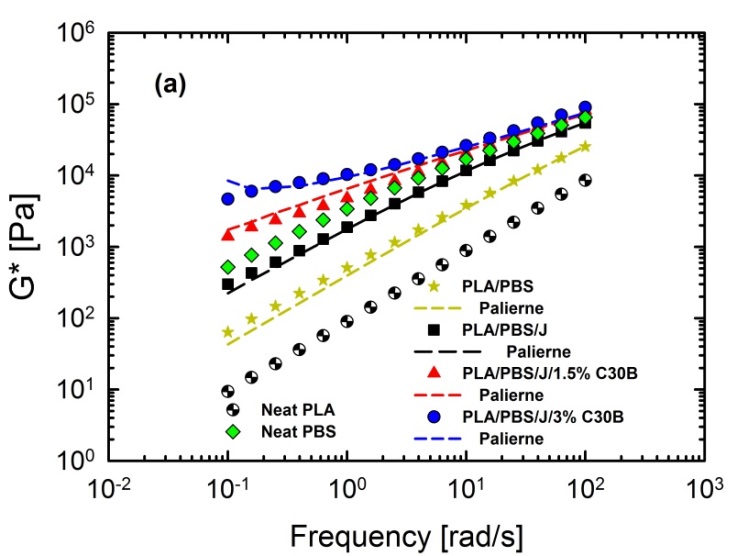 | 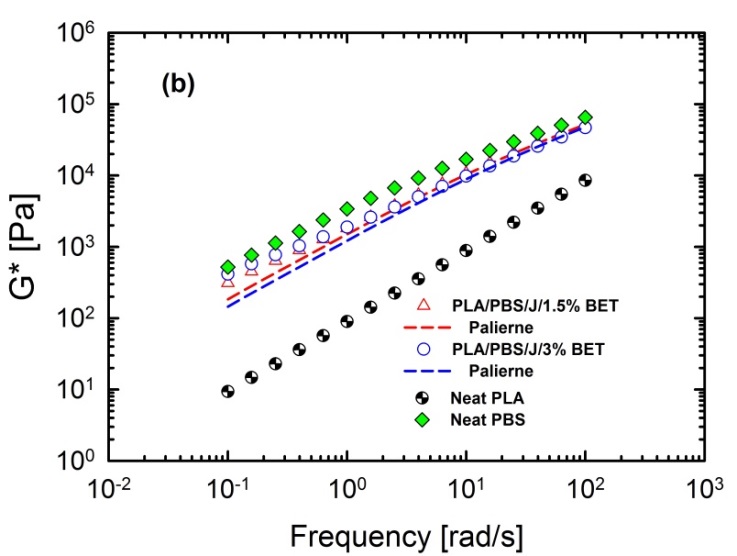 |
| --- | --- |

**Figure S1.** Palierne model fits for (**a**) PLA/PBS and PLA/PBS/J blends and PLA/PBS/J/C30B and (**b**) PLA/PBS/J/BET blend nanocomposites. BET is Betsopa™, C30B is Cloisite^®^30B, and J is Joncryl.

The discrepancies between the experimental and estimated results could be attributed the fact that this model is a simplistic emulsion model and with increasing organoclay content the matrix becomes increasingly concentrated. It can also be seen that the results in case of the BET-filled blends are even less satisfactory, owing to the broadly dispersed phase distribution. Tao et al. [2] discussed that size reduction in nanoparticle induced blends where nanoparticles are trapped at the interface is not due to the effect of lowering interfacial tensions as in the case of classical copolymer compatibilizers but rather suppressing coalescence due to formation of a rigid shield around the dispersed phase. The interfacial tension values obtained from these fits are listed in Table S1.

**Table S1.** Interfacial tension predicted by the Palierne model. BET is Betsopa™, C30B is Cloisite^®^30B, and J is Joncryl. The Palierne model might not be perfectly suitable for analysing these blends and blend nanocomposites; however, it could be used for the sake of estimation.

| **Sample** | $\boldsymbol{\gamma[}\boldsymbol{mN}/{\boldsymbol{m}^{\boldsymbol{2}}}\boldsymbol{]}$ |
| --- | --- |
| PLA/PBS | 29.8 |
| PLA/PBS/J | 15.7 |
| PLA/PBS/J/1.5%C30B | 4.8 |
| PLA/PBS/J/3%C30B | 3.7 |
| PLA/PBS/J/1.5%BET | 13.01 |
| PLA/PBS/J/3%BET | 11.37 |

These results show that adding Joncryl and organoclays reduces interfacial tensions; however, C30B is more efficient in reducing the blend interfacial tensions compared with BET. This is also consistent with morphological results, which indicate that surfaces of PBS droplets in C30B-filled blends are smooth compared with rough surfaces in BET-filled blends, insets of Figure 2c,f.

References

1. Palierne, J. F. Linear rheology of viscoelastic emulsions with interfacial tension*.* *Rheol. Acta* **1990**, *29*, 204−214.
2. Tao, F.; Auhl, D.; Baudouin, A-C.; Stadler, F. J.; Bailly, C. Influence of multiwall carbon nanotubes trapped at the interface of an immiscible polymer blend on interfacial tension. *Macromol. Chem. Phys.* **2013**, *214*, 350-360.

© 2017 by the authors. Submitted for possible open access publication under the
terms and conditions of the Creative Commons Attribution (CC BY) license (http://creativecommons.org/licenses/by/4.0/).
